# Clinical Confirmation of Pan-Amyloid Reactivity of Radioiodinated Peptide ^124^I-p5+14 (AT-01) in Patients with Diverse Types of Systemic Amyloidosis Demonstrated by PET/CT Imaging

**DOI:** 10.3390/ph16040629

**Published:** 2023-04-21

**Authors:** Emily B. Martin, Alan Stuckey, Dustin Powell, Ronald Lands, Bryan Whittle, Craig Wooliver, Sallie Macy, James S. Foster, Spencer Guthrie, Stephen J. Kennel, Jonathan S. Wall

**Affiliations:** 1 Department of Medicine, University of Tennessee Graduate School of Medicine, Knoxville, TN 37920, USAjfoster3@utk.edu (J.S.F.); skennel@utmck.edu (S.J.K.); jwall@utmck.edu (J.S.W.); 2 Department of Radiology, University of Tennessee Medical Center, Knoxville, TN 37920, USA; 3 Attralus Inc., South San Francisco, CA 94133, USA

**Keywords:** amyloidosis, ^124^I-p5+14, p5+14 peptide, AT-01, systemic amyloidosis, PET/CT imaging

## Abstract

There are at least 20 distinct types of systemic amyloidosis, all of which result in the organ-compromising accumulation of extracellular amyloid deposits. Amyloidosis is challenging to diagnose due to the heterogeneity of the clinical presentation, yet early detection is critical for favorable patient outcomes. The ability to non-invasively and quantitatively detect amyloid throughout the body, even in at-risk populations, before clinical manifestation would be invaluable. To this end, a pan-amyloid-reactive peptide, p5+14, has been developed that is capable of binding all types of amyloid. Herein, we demonstrate the ex vivo pan-amyloid reactivity of p5+14 by using peptide histochemistry on animal and human tissue sections containing various types of amyloid. Furthermore, we present clinical evidence of pan-amyloid binding using iodine-124-labeled p5+14 in a cohort of patients with eight (*n* = 8) different types of systemic amyloidosis. These patients underwent PET/CT imaging as part of the first-in-human Phase 1/2 clinical trial evaluating this radiotracer (NCT03678259). The uptake of ^124^I-p5+14 was observed in abdominothoracic organs in patients with all types of amyloidosis evaluated and was consistent with the disease distribution described in the medical record and literature reports. On the other hand, the distribution in healthy subjects was consistent with radiotracer catabolism and clearance. The early and accurate diagnosis of amyloidosis remains challenging. These data support the utility of ^124^I-p5+14 for the diagnosis of varied types of systemic amyloidosis by PET/CT imaging.

## 1. Introduction

Systemic amyloidosis is a multi-organ disease wherein misfolded proteins deposit as fibrils in the extracellular spaces of tissues, resulting in progressive organ dysfunction and severe morbidity [1]. Amyloid deposits are composed of structurally and functionally diverse precursor proteins [2] associated with extracellular matrix components and serum components, including heparan sulfate proteoglycans (HSPG), the serum amyloid P component (SAP), and apolipoproteins [3].

The most common types of systemic amyloidosis diagnosed in the USA result from the deposition of monoclonal immunoglobulin light chains (AL), variant or wild-type transthyretin (ATTRv or ATTRwt), or leukocyte chemotactic factor-2 (ALECT2). The rarer types of systemic amyloidosis are invariably hereditary, resulting from germline mutations that render typically benign proteins amyloidogenic [1]. Patients with systemic amyloidosis are generally present with a heterogeneous anatomic distribution of amyloid and often with combinations of organ involvement. Cardiac and renal amyloid deposits are common in AL amyloidosis and result in morbidity and mortality in these patients [4]. Deposition of amyloid in other organs and tissues may cause clinical sequelae or remain clinically silent due to the early stage of deposition (presymptomatic deposits) and the lack of detectable serum biomarkers. The contribution of these deposits to quality of life and mortality remains unclear. However, studies have shown that the presence of amyloid in more than one organ is associated with poorer outcomes [5,6,7,8]. The diverse clinical presentation of amyloidosis, which often shares overlapping symptomology with more common disorders, makes rapid and accurate diagnosis challenging [9]. The lack of imaging diagnostics to detect multiple types of amyloidosis and key abdominothoracic organ involvement is a significant unmet need that contributes to delayed diagnosis and an often-incomplete appreciation of the amyloid burden. Therefore, a sensitive, facile, and non-invasive method for detecting the systemic distribution of diverse types of amyloid in patients is of considerable clinical benefit.

Molecular imaging can provide quantitative, non-invasive methods for the detection of amyloid throughout the body. At present, patients in the UK and Netherlands with diverse types of systemic amyloidosis are being assessed clinically using planar gamma scintigraphic imaging with iodine-123-labeled SAP [10,11]. As a natural component of amyloid, ^123^I-SAP partitions into amyloid deposits and can be readily detected in abdominothoracic organs with the notable exception of the heart [12]. More recently, bone-seeking agents such as technetium-99m pyrophosphate (^99m^Tc-PyP) and ^99m^Tc- 3,3-diphosphono-1,2-propanodicarboxylic acid (DPD) have been employed to detect cardiac deposits in select patients with ATTR associated amyloidosis who have been confirmed not to have AL [13,14]. Despite their increasing utility for diagnosing and monitoring changes in amyloid load, each of these radiotracers has limitations in determining whole-body amyloid load quantitatively and detecting amyloid in all types of amyloid and key abdominothoracic organs.

To address the limitations of the current radiotracers used for amyloid imaging, we have characterized a synthetic peptide that is capable of binding specifically to all types of amyloid. This broad, but specific, reactivity is mediated by multivalent electrostatic interactions of the peptide with dense negative charge arrays present on both the hypersulfated HSPG and proteinaceous fibrils in the amyloid mass [15]. The peptide p5+14 is a synthetic, polybasic, 45 amino acid peptide that forms an alpha helix in the presence of dense electronegative surfaces, such as those abundantly available in amyloid [15]. In its proposed helical form, the lysine residues of the peptide are predicted to align on one face of the peptide, thereby supporting a specific electrostatic interaction with the linear array of negative charges present along the long axis of the amyloid fibril and charged glycosaminoglycans. Herein, we demonstrate through peptide histochemistry on both human and animal-derived tissue sections the ability of biotinylated p5+14 to specifically bind multiple types of amyloid. This mode of interaction indicates that peptide p5+14 is capable of binding all amyloid types, regardless of the precursor protein from which the amyloid deposits are formed.

We have previously demonstrated the peptide’s in vivo amyloid-targeting capability in a murine model of systemic amyloidosis using small animal SPECT/CT and PET/CT imaging [15,16,17,18,19]. The promising in vivo data, coupled with its established potential for pan-amyloid binding, led to the development and evaluation of p5+14 as a radiotracer in the clinical setting. Our preclinical data supported the use of iodine-124-labeled peptide as an amyloid-targeting radiotracer due to its relatively long half-life (4.2 days) and the inherent biological properties of dehalogenation, which allowed for visualization of renal amyloid once unbound radiotracer had been catabolized and excreted [17].

In this report, a subset of data from a single site, first-in-human evaluation of iodine-124-labeled-p5+14 (AT-01; INN designation iodine-124-evuzamitide) for the detection of diverse types of systemic amyloidosis by PET/CT imaging, is discussed. Herein, we highlight the pan-amyloid reactivity and multi-organ distribution of ^124^I-p5+14 in a subset (*n* = 8) of the enrolled patients (*n* = 52) who presented with biochemically distinct amyloid types, including light chain (ALκ and ALλ), transthyretin (ATTRv and ATTRwt), leukocyte chemotactic factor-2 (ALECT2), gelsolin (AGel); lysozyme (ALys), and apolipoprotein-A1 (AApoA1).

## 2. Results

### 2.1. Peptide Histochemistry

**Figure 1:**
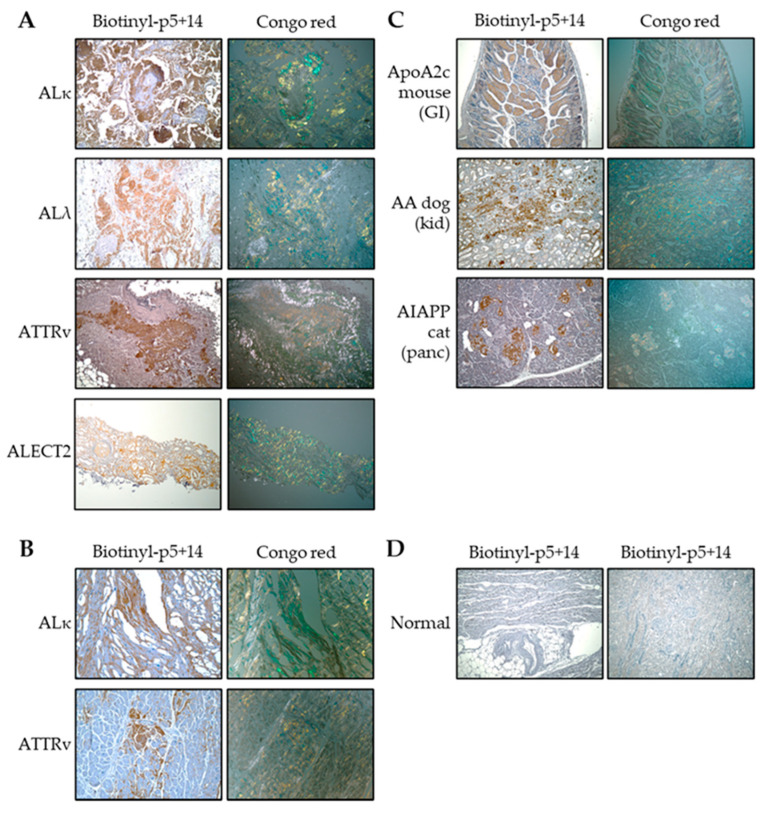
Peptide histochemistry and Congo red analysis of tissue sections demonstrate the pan-amyloid reactivity of p5+14. The brown-colored stain of biotinyl-p5+14 can be seen in (**A**) patient-derived renal tissues, magnification 10×; (**B**) patient-derived cardiac tissues, magnification 20×; and (**C**) animal-derived tissues, magnification 10×. The peptide colocalized to Congophilic regions within tissue, as demonstrated with green–gold birefringence in consecutive tissue sections. (**D**) Normal heart (left) and kidney (right) tissues were negative with biotinyl-p5+14, magnification 10×.

The pan-amyloid reactivity of biotinylated peptide p5+14 was demonstrated using a panel of formalin-fixed human or animal tissue sections from various organs containing distinct amyloid types. The tissue amyloids analyzed included AL kappa, AL lambda, ATTRv, ALECT2, apolipoprotein-A2c (ApoA2c), serum amyloid A (AA), and islet amyloid polypeptide (AIAPP), all of which stained positive with biotinyl-p5+14 (Figure 1). In contrast, normal human cardiac and renal tissue was not immunostained. These data support our hypothesis that peptide p5+14 binds all types of amyloid derived from various anatomic sites.

### 2.2. PET/CT Imaging with ^124^I-p5+14

**Figure 2:**
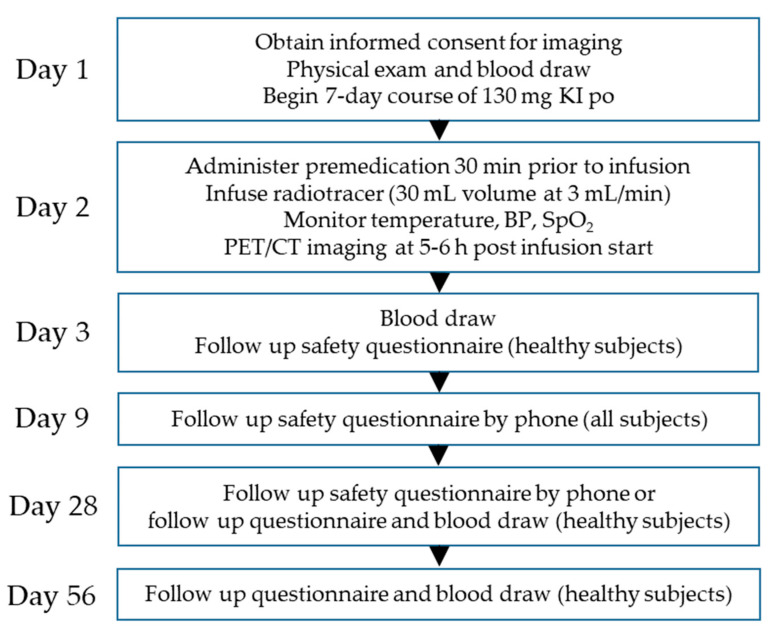
Phase 1/2 study design. Participants were asked to begin a 7-day course of potassium iodide (KI; iOSAT, ANBEX INC., Livingston, NJ) to reduce thyroid exposure to radioactivity. Prior to infusion, participants were given acetaminophen (650 mg) and diphenhydramine (or a suitable alternative; 25 mg). Vital signs were monitored prior to, during, and for 50 min after the infusion of ^124^I-p5+14.

The Phase 1/2 clinical trial evaluating the safety and efficacy of ^124^I-p5+14 in patients and healthy volunteers was conducted at a single site over 3 years, ending in 2021. Initially, the medical records of the patients were obtained after signed medical releases were provided. The records were then reviewed to assess the diagnosis of amyloidosis and collect data describing the known or anticipated organ-based distribution of amyloid based on biopsies, imaging, biomarkers, and physical examinations reported by the treating physicians. Following this prescreen, patients traveled to Knoxville, TN, where written consent was obtained, followed by a physical exam and phlebotomy. Subjects also began a 7-day course of 130 mg potassium iodide (KI). On day 2, subjects were administered antihistamine and acetaminophen before receiving 74 MBq (<2 mg peptide) ^124^I-p5+14 intravenously by slow infusion (3 mL/min for 10 min). PET/CT images were acquired at 5 h post-infusion. The optimal time for imaging was established in the first three patients as part of assessing dosimetry for safety. It was determined that five to six hours post-infusion allowed for visualization of renal amyloid and would be appropriate for whole-body imaging. Safety assessments and follow-ups for patients occurred on days 9 and 28, and for healthy volunteers, on days 3, 28, and 56 (Figure 2). This report describes the data collected on a cohort of eight patients with diverse types of systemic amyloidosis to assess the pan-amyloid reactivity of the radiotracer and five healthy volunteers who were enrolled. Table 1 displays participant characteristics and radiopharmaceutical details for the dose each subject received.

**Figure 3:**
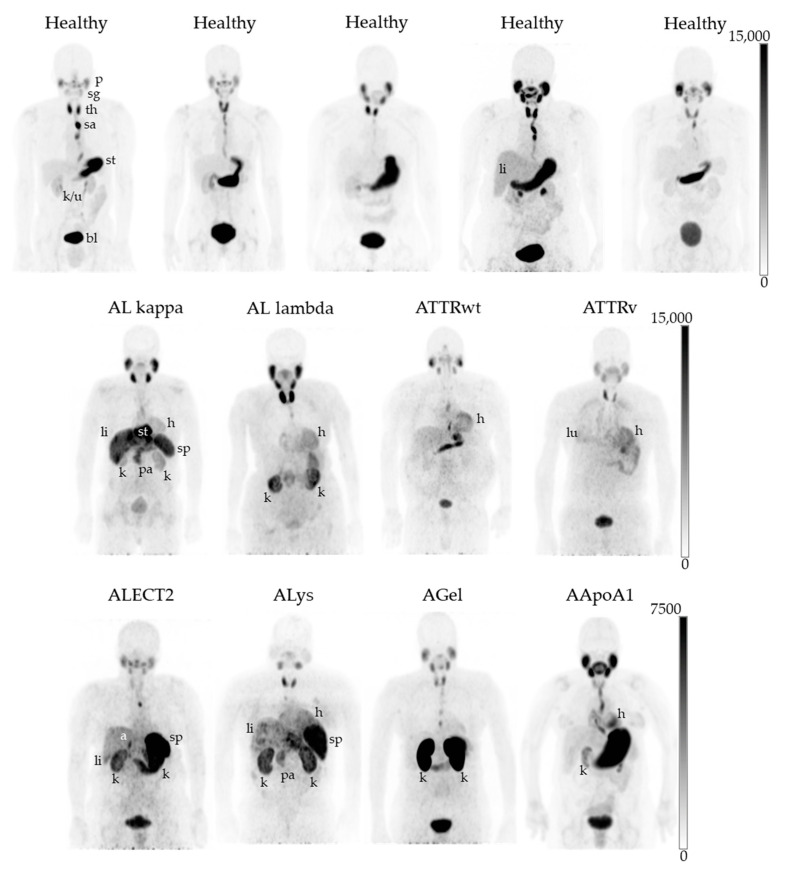
Biodistribution of ^124^I-p5+14 in healthy subjects and patients with diverse types of systemic amyloidosis. Maximum intensity projection PET images of healthy subjects (healthy) and patients with amyloidosis (immunoglobulin light chain kappa and lambda (ALκ and ALλ), transthyretin wild type and variant (ATTRwt and ATTRv), leukocyte chemotactic factor 2 (ALECT2), lysozyme (ALys), gelsolin (AGel), and apolipoprotein A1 (AApoA1)-associated amyloidoses). All subjects were administered 74 MBq (+/− 10%) I-124, and images were scaled to a minimum and maximum of 0–15,000 Bq/cc, except for the ALECT2 patient, who received 38.5 MBq, where the image is scaled to 7500 Bq/cc. For clarity, due to its atypical location, the stomach of the AL kappa patient has been labeled but does not indicate amyloid uptake in this organ. P: parotid gland; sg: salivary gland; th: thyroid gland; sa: saliva; st: stomach; k/u: kidney/ureter; bl: urinary bladder; h: heart, li: liver; sp: spleen; k: kidney; pa: pancreas; lu: lung; a: adrenal gland.

In healthy subjects, five hours after a single IV infusion of ^124^I-p5+14, radioactivity was universally observed in the parotid and salivary glands, thyroid gland, saliva (as a bolus in the esophagus), stomach lumen, renal pelvis, and ureter, and urinary bladder. However, there was no significant retention of ^124^I-p5+14 in the abdominothoracic organs of healthy subjects, with one singular exception (shown as the fourth healthy subject in Figure 3), in whom renal and diffuse hepatic radioactivity was deemed to be modestly higher than background tissues.

In patients with amyloidosis, radioactivity was observed in diverse abdominothoracic organs, including the heart, liver, spleen, kidneys, pancreas, and lungs (Figure 3).

**Figure 4:**
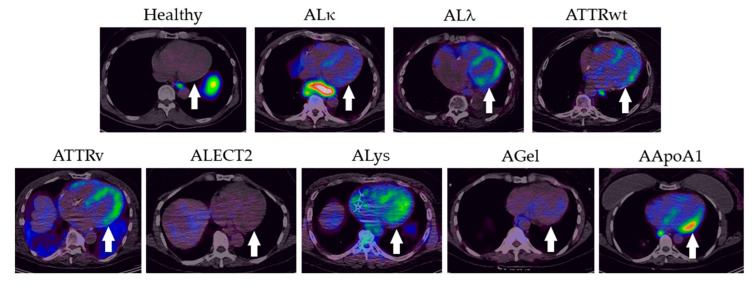
Transaxial images of the heart from a representative healthy subject and patients with diverse forms of systemic amyloidosis following injection of ^124^I-p5+14. Retention of radiotracer in the left ventricular wall (arrow) was seen in all subjects except the healthy individual and patients with ALECT2 and AGel amyloidosis. Images have been scaled as described in Figure 3.

Myocardial uptake of ^124^I-p5+14, when present, principally involved the left ventricle, including the interventricular septum and the posterior wall. However, the right ventricular and atrial walls were also imaged in some patients with AL and ATTR, as well as the ALys patient and the AApoA1 patient (Figure 4). In contrast, only trace blood pool radioactivity was observed in the ventricular lumen of patients with ALECT2, AGel, and healthy subjects. This suggests the absence of amyloid due to the lack of radiotracer accumulation in the myocardium (Figure 4).

**Figure 5:**
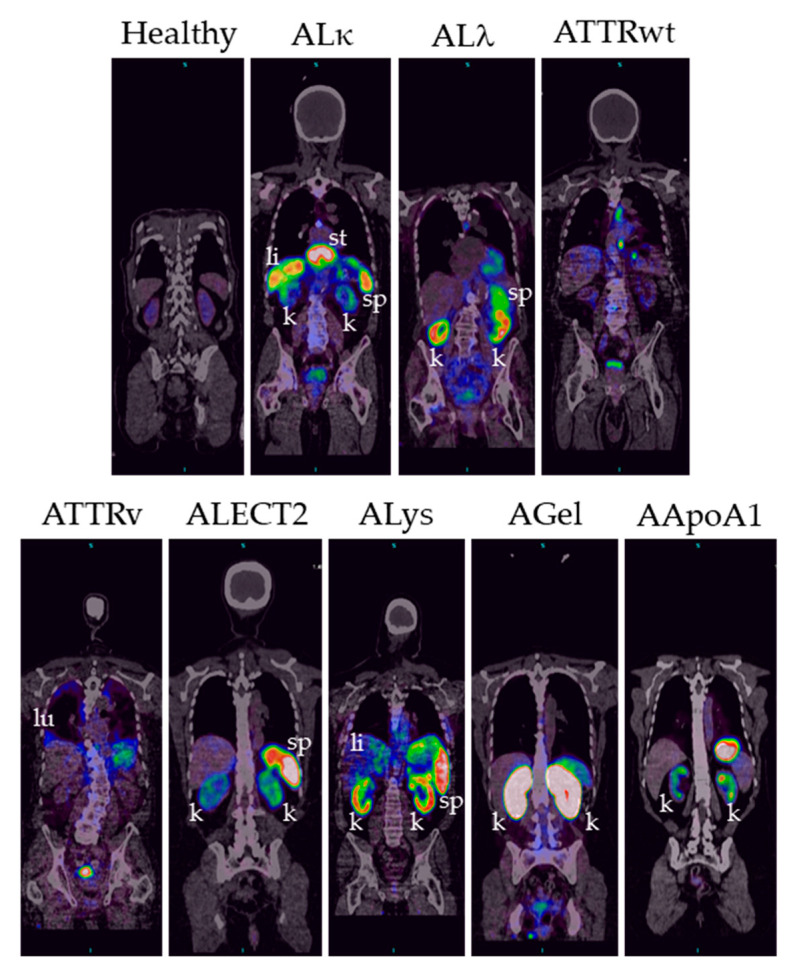
Coronal PET/CT images of a representative healthy subject and patients showing specific and heterogenous uptake of radiotracer in abdominothoracic organs. Images have been scaled as described in Figure 3.

Extracardiac amyloid deposits, including those in the liver, spleen, kidney, and lung, can be appreciated with single-slice, fused PET/CT images, as shown in Figure 5. These images highlight the heterogeneity of amyloid deposition in patients with various types of systemic amyloidosis.

The comparison of organ-associated amyloid in the patients presented in this report, based on observations made in the medical record, and the distribution of ^124^I-p5+14 accumulation as reported after careful review of each organ, revealed excellent concordance for major abdominothoracic organs (Table 2). In each of the eight patients, imaging not only confirmed amyloid in the clinically suspected organs but also revealed the accumulation of radioactivity in organs not appreciated clinically. For the AGel patient, amyloid in the nerve and skin was the clinical presentation; however, these tissues were not routinely imaged using this protocol, likely due to the low amyloid load in these tissues.

## 3. Discussion

There are 42 types of pathologic amyloidosis recognized by the International Society of Amyloidosis, and of these, 20 are defined as systemic disorders. ATTR and AL are the most common forms of systemic amyloidosis. Treatment for both ATTR and AL currently focuses on the prevention of further amyloid accumulation. For patients with ATTR, silencers are approved to lower the production of transthyretin in the liver using oligonucleotides and small interfering RNA [20,21]. Alternatively, the dissociation of the transthyretin tetramer, a prerequisite for amyloid formation, can be halted using stabilizers such as tafamidis [22,23,24]. In patients with AL-associated amyloidosis, light chain production by the plasma cell clone is inhibited using proteasome inhibitors, chemotherapy, or anti-plasma cell immunotherapy using daratumumab [25]. In patients with rare types of amyloid, where renal deposition is generally the most common pathologic feature, treatment options are limited or not available. Currently, there is no standard way to monitor changes in amyloid load in practice. Monitoring disease burden with progression, regression, and/or changes in amyloid load during clinical trials of therapeutics, as they become available, would be invaluable.

Systemic amyloidosis is characterized by heterogeneous anatomic depositions of amyloid. Not recognizing the extent of the whole-body amyloid burden can hinder a complete appreciation of the pathology and its sequelae. Whole-body imaging can provide a complete picture of amyloid distribution; however, there are currently no FDA-approved agents available for this purpose. The radioiodinated SAP component (^123^I-SAP) binds all types of amyloid and has been used extensively in the UK and the Netherlands to image extracardiac amyloid. Imaging of cardiac ATTR-associated amyloidosis with bone-seeking ^99m^Tc-PYP or ^99m^Tc-DPD is routinely performed, but these reagents do not bind amyloid directly but rather accumulate in areas of the heart with microcalcifications [26]. Additionally, ^18^F-florbetapir detects various amyloid types but has low affinity for TTR, cannot image hepatic amyloid, and does not accurately detect renal deposits [27]. Other pan-amyloid-binding biologicals are well described [28,29,30,31,32,33,34,35], many of which use pattern recognition motifs for binding rather than primary sequence determinants [31,32,33]. The peptide p5+14 directly binds amyloid-associated fibrils and hypersulfated HSPG via multivalent electrostatic interactions with the negative charges presented by fibrils and amyloid-associated glycans. Accordingly, we posit that ^124^I-p5+14 falls within the pattern recognition family of amyloid-reactive reagents [15].

Early preclinical development of the radiotracer utilized direct radioiodination of the lone tyrosine residue at position four of the p5+14 peptide. This circumvented the need for clinical chelators, which would involve chemical modification of one of the 12 lysine side chains that are critically important for amyloid binding or incorporation of a cysteine residue to enable site-specific bifunctional chelator attachment [36]. Moreover, the preclinical radioiodination method was readily adaptable for iodine-124 or iodine-123 and therefore represented a facile development path for either PET or SPECT imaging. Amyloidosis is a systemic disease in which the organ-specific amyloid load, notably in the heart and kidney, has prognostic implications [4,37]. However, extra-cardiorenal amyloid may impact the quality of life and produce clinical manifestations that are not appreciated due to our inability to detect amyloid in all anatomic sites. Therefore, PET/CT imaging using iodine-124 was the imaging modality of choice, allowing quantitative, high-resolution visualization of the distribution of amyloid throughout the body.

Iodine-124 is an exotic isotope, so called because it is not routinely used for clinical PET/CT imaging but is well suited for immunoPET imaging [38,39,40,41,42,43], or in this case, peptide PET imaging [44]. Iodine-124 is a cyclotron-produced radionuclide with a 4.2 day half-life, which affords advantages in the production and shipping of ^124^I-p5+14 from a single site. The radionuclide decays to tellurium-124 by electron capture with a 25.6% positron emission [45]. Although iodine-124 has a similar positron range (Rmean = 4.4 mm) to gallium-68 (~4 mm), it is greater than fluorine-18 (0.6 mm) or Zr-89 (1.3 mm) [45], which can lead to degradation of high-resolution PET images. However, high-resolution PET data using iodine-124 is possible using prompt gamma correction and point spread function reconstruction methods and can be further improved with correction for the positron range [46].

During the early development of p5+14 as a PET radiotracer, other radionuclide alternatives were considered. At that time, methods for incorporating F-18 into peptides were limited and inefficient, and thus, fluorination of p5+14 was not considered a viable alternative. Similarly, zirconium-89 labeled p5+14 was not evaluated because the clinically used chelator, deferoxamine-pPhe-NCS, would utilize one of the critical lysine side chains in the amyloid binding domain of the peptide.

In addition to the physical characteristics of I-124 as a suitable radionuclide for PET imaging of the peptide p5+14, the biological properties are also favorable. In patients with systemic amyloidosis, accumulation of amyloid in the heart and kidneys is one of the leading causes of mortality and a reduction in quality of life. Therefore, the detection of amyloid in both organs in a single imaging procedure was deemed an important feature of an amyloid imaging agent. Imaging pathology in the organ through which a radiotracer is catabolized is challenging, if not impossible, in most cases [47]. However, we demonstrated using amyloid-free mice that the radioactivity in the kidneys rapidly decreased following the initial appearance after IV injection of radioiodinated p5+14 [17]. Therefore, we find significant advantages in using the non-residualized radioiodide as compared to a residualized nuclide, e.g., Zr-89, for imaging renal amyloid deposits. We have hypothesized that radioiodide clearance from the kidney is due to the action of intracellular dehalogenase enzymes that strip the radioiodide from the peptide. In contrast, when the radiotracer was bound to extracellular renal amyloid deposits in mice with severe systemic AA amyloidosis, it was protected from dehalogenation and remained at the amyloid site, detectable even up to seven days post-injection [17]. Thus, PET imaging of ^124^I-p5+14 at~ 5 h post-injection can confidently be used to specifically detect renal amyloid in human subjects.

Herein, we have described the pan-amyloid reactivity of a novel peptide radiotracer capable of imaging, in patients, diverse types of amyloidosis throughout the body, including the heart. In healthy subjects, comparatively little retention of ^124^I-p5+14 was observed in the abdominothoracic organs. The physiological distribution of radioactivity was limited to the renal pelvis, ureters, and bladder, as well as the salivary and parotid glands, thyroid, and stomach lumen (Figure 2). ^124^I-p5+14 was rapidly cleared from the circulation and accumulated in amyloid-laden abdominothoracic organs due to its specific binding to two ubiquitous amyloid components, fibrils and HSPG, and functional dehalogenation.

Despite the small number of patients with rarer types of amyloidosis assessed in this early-phase study, the images are consistent with the presentation of the disease reported in the clinical record and with reports in the literature. In addition, organs that were not appreciated clinically as containing amyloid were imaged using ^124^I-p5+14. Amyloid in these anatomic sites may represent subclinical deposits or those with yet unknown clinical sequelae. Recent autopsy studies do indicate that amyloid is often widespread throughout the body, consistent with ^124^I-p5+14 imaging [48,49,50].

An accurate and prompt diagnosis of amyloidosis remains a challenge in the clinical setting. The heterogeneous clinical presentation and related diversity of amyloid-related disorders are contributors to this. PET/CT imaging with ^124^I-p5+14 can provide a facile, non-invasive method for clinical diagnosis and disease staging for patients with any type of systemic amyloidosis. Further assessment of ^124^I-p5+14 PET/CT imaging is warranted to more fully demonstrate its clinical utility for patients with systemic amyloidosis.

## 4. Materials and Methods

### 4.1. Peptide Histochemistry and Congo Red Staining

The peptide p5+14 (with a cysteine residue at the N-terminal) was prepared for tissue staining by biotinylation, according to the manufacturer’s instructions, using a maleimide-biotin conjugation kit (Pierce, Grand Island, NY, USA). Six μm-thick formalin-fixed, paraffin-embedded human or animal amyloid-laden tissue sections were deparaffinized, placed on slides, and incubated in citrate antigen retrieval solution (Citrus Plus; BioGenex, Fremont, CA, USA) at 90 °C for 30 min. The biotinylated peptide p5+14 was added at a concentration of 5 μg/mL in PBS and incubated overnight at 4 °C in a humidified chamber. The slides were developed using the Vectastain Elite ABC development kit (Vector Labs, Burlingame, CA, USA) and visualized using diaminobenzidene (Vector Labs).

Detection of amyloid was achieved in consecutive tissue sections by staining with an alkaline Congo red solution (0.8% *w/v* Congo red, 0.2% *w/v* KOH, 80% ethanol) for 1 h at room temperature, followed by a counterstain with Mayer’s hematoxylin for 2 min. All tissue sections were examined using a Leica DM500 light microscope (Leica, Wetzlar, Germany) fitted with cross-polarizing filters (for Congo red). Digital microscopic images were acquired using a cooled CCD camera (SPOT; Diagnostic Instruments, Sterling Heights, MI, USA).

### 4.2. Study Participants

A total of 57 participants were administered ^124^I-p5+14 in this study; however, this report highlights the biodistribution of radiotracer uptake in a subset of eight patients, each with a distinct type of systemic amyloidosis (Table 1). The mean age for this subset was 65.4 ± 4.5 y, and the median time from diagnosis was 4.5 y (IQR: 2.3–5.8). Additionally, 5 healthy subjects (2 M/3 F) with a mean age of 57.6 ± 8.2 y (IQR: 50–65) served to assess the normal physiological distribution of the radioactivity.

### 4.3. Peptide and Radiolabeling

The peptide p5+14 with the amino acid sequence: GGGYS KAQKA QAKQA KQAQK AQKAQ AKQAK QAQKA QKAQA KQAKQ was manufactured under GMP conditions by AmbioPharm Inc. (North Augusta, SC), supplied as a lyophilized powder in 3 mg aliquots, and stored at −20 °C. Iodine-124 was purchased from 3D Imaging (Little Rock, AR 72205; DMF# 025853). Single patient doses of ^124^I-p5+14 were prepared on the day of use with soluble iodogen as the oxidant [51]. Following tests to ensure quality (>90% radiopurity, >90% peptide purity, bioactivity, and non-pyrogenicity), the subjects received a single intravenous infusion of the radiotracer (74 ± 0.7 MBq I-124 and no more than 2 mg of peptide) at 3 mL/min for 10 min [51].

### 4.4. Study Design

This is a post hoc analysis of the Phase 1/2 clinical trial of ^124^I-p5+14 (AT-01) to assess safety and amyloid-reactivity in patients with a confirmed diagnosis of systemic amyloidosis [51]. The anatomic distribution of amyloid was assessed from the patients’ medical records prior to imaging. Healthy subjects had no evidence of amyloid-related pathologies and no family history of hereditary amyloidosis. No participants were taking heparin or heparin-derived anticoagulants. The study design is shown in Figure 1.

### 4.5. Image Acquisition

PET/CT images were acquired using a Siemens PET/CT Biograph imaging platform (Siemens, Knoxville, TN) with a low-dose CT (120 kVp, 50 effective mAs) at ~5 h post-infusion using 5 min PET acquisitions per bed position.

PET data were reconstructed using a 3DOSEM algorithm with attenuation weighting and prompt gamma correction with a 168 × 168 image matrix and an image resolution of ~8 mm full-width half maximum. CT data were reconstructed using a medium smoothing kernel and 4 mm reconstruction increments.

### 4.6. Image Analysis

PET/CT images were visually evaluated for organ-specific retention of radioactivity using the XD General Oncology Review application in Mirada Medical DBx (Build 1.2.0.59) by a nuclear medicine physician blinded to subjects’ disease status. Maximum intensity projections (MIPs) and PET/CT images were prepared using Inveon Research Workplace (IRW) software (Ed. 4.2 [4.2.0.15], Siemens Preclinical Solutions).

### 4.7. Study Oversight

The study protocol was approved by the US Food and Drug Administration and performed under the auspices of Investigational New Product (IND) No. 132282. Approval was obtained from the Institutional Review Board (protocol #4386) at the UT Graduate School of Medicine (Knoxville, TN, USA). All participants provided informed written consent prior to the prescreening of medical records and study participation.

## Figures and Tables

**Table 1:** Patient cohort and healthy subject characteristics with dose information.

Subject	Amyloid Type	Sex	Age (y)	Time from Diagnosis (y)	Injected Dose (MBq)	Injected Peptide (mg)
P05	ALECT2	F	63	6	38.5 ^1^	1.30
P07	ALκ	M	72	3	74.4	1.37
P16	ALλ	F	79	5	74.4	1.34
P23	ALys	M	45	4	74.9	1.10
P24	AGel	F	63	16	72.5	1.52
P26	ATTRwt	M	77	2	73.4	1.48
P30	AApoA1	F	49	5	75.9	1.27
P32	ATTRv	M	75	2	76.2	1.20
P43	HV	M	68	NA	74.5	1.40
P44	HV	F	52	NA	74.8	1.40
P45	HV	F	47	NA	73.6	1.62
P47	HV	F	60	NA	74.0	1.52
P54	HV	M	61	NA	73.3	1.63

^1^ Patient was imaged before the maximum dose was determined based on dosimetry calculations. HV, healthy volunteer; NA, not applicable.

**Table 2:** Clinical amyloid distribution and ^124^I-p5+14 sites of accumulation by imaging.

Organ	AL kappa		AL lambda		ATTRwt		ATTRv		ALECT2		ALys		AGel		AApoA1	
	C ^1^	I	C	I	C	I	C	I	C	I	C	I	C	I	C	I
Heart	✓ ^2^	✓		✓	✓	✓	✓	✓			✓	✓				✓
Lung				✓				✓								
Liver	✓	✓				✓		✓		✓	✓	✓				
Spleen		✓				✓		✓		✓	✓	✓				
Kidney		✓	✓	✓		✓		✓	✓	✓	✓	✓		✓	✓	✓

^1^ C, clinical distribution of amyloid based on medical record; I, imaging distribution of amyloid based on ^124^I-p5+14 uptake. ^2^ Positive uptake of the radiotracer (✓) is the result of a stringent, clinical read by a nuclear medicine physician using appropriate visualization software and window parameters for each organ. Therefore, this distribution may not be obvious in the MIP images (Figure 3), which are not threshold dependent, nor the coronal PET/CT images (Figure 5), which capture the distribution of radioactivity in a single slice.

## Data Availability

Not applicable.
